# Mapping the Mind: A Network Meta-Analysis of Mindfulness and Traditional and Digital Interventions for Cognitive and Behavioral Enhancement in Children With Attention-Deficit/Hyperactivity Disorder (ADHD)

**DOI:** 10.7759/cureus.102453

**Published:** 2026-01-28

**Authors:** Hosam Hadi Hassan Awaji, Rawan A Alharbi, Manal B Mokli, Ghadh M Balawi, Yahya S ALzahrani, Alaa R Khan, Tebra A Bima, Maha G Atwie, Amal O Alatwai, Sarah A Alatawi, Rawan M Albalawi

**Affiliations:** 1 Preventive Medicine, North West Armed Forces Hospitals, Tabuk, SAU; 2 Nursing, King Fahad Armed Forces Hospital, Jeddah, SAU; 3 Nursing: Preventive Medicine, North West Armed Forces Hospitals, Tabuk, SAU; 4 Nursing Family Medicine, North West Armed Forces Hospitals, Tabuk, SAU; 5 Radiology, King Fahad Armed Forces Hospital, Jeddah, SAU; 6 Critical Nursing Care, King Fahad Armed Forces Hospital, Jeddah, SAU; 7 Nursing: Family Medicine, North West Armed Forces Hospitals, Tabuk, SAU; 8 Nursing, North West Armed Forces Hospitals, Tabuk, SAU; 9 Family Medicine, North West Armed Forces Hospitals, Tabuk, SAU

**Keywords:** adhd, behavioral parent training, brain function enhancement, cognitive behavioral therapy, mindfulness-based therapy, working memory training

## Abstract

Pharmacological and behavioral interventions are commonly used to manage attention-deficit/hyperactivity disorder (ADHD). This disorder is highly prevalent among children and adolescents. New non-pharmacological approaches, including mindfulness-based therapies (MBT), neurofeedback, and working memory training (WMT), may represent potential alternatives. This network meta-analysis aims to assess the comparative effectiveness of various nonpharmacological interventions for pediatric ADHD. Electronic databases were searched to identify clinical trials that compared different treatments in ADHD to each other, usual care, or waiting list from inception to March 22, 2025. Sixteen studies representing 806 participants were included in this study. The network compared several intervention types: MBT, neurofeedback, cognitive-behavioral therapy (CBT), WMT, and behavioral parent training (BPT) versus control or active comparators. Primary outcome measures were child behavior checklist (CBCL), Conners' rating scale (CRS), inattention and impulsivity measurement, ADHD rating scale-IV, SWAN (strengths and weaknesses of ADHD symptoms and normal behaviors), and parenting stress index-short form (PSI-SF). The ranking of treatment efficacy was based on the surface under the cumulative ranking curve (SUCRA). Family MBT was the most effective intervention for reducing CBCL scores (mean difference (MD) = -5.49, 95% confidence interval (CI): -8.65 to -2.33, p = 0.001) and inattention symptoms (MD = -8.88, 95% CI: -13.50 to -4.26, p < 0.001), ranking highest in effectiveness. BPT (face-to-face) showed the greatest improvement in CRS scores, though not statistically significant (MD = -6.15, 95% CI: -14.49 to 2.19, p = 0.148). Concerning parenting and family outcomes, BPT had the highest probability (65.9%) of reducing parental stress, while usual care was the least effective (69.2%). SUCRA values indicated MBT interventions (family MBT, online MBP, family-based mindfulness intervention (FBMI)) consistently ranked among the most effective treatments for ADHD symptoms, while control groups consistently ranked lowest. This network meta-analysis supports MBT and BPT as effective non-pharmacological treatments for ADHD. Family MBT was particularly effective for inattention and hyperactivity, while BPT significantly reduced parenting stress. However, the findings need to be confirmed through further research with larger sample sizes and longer follow-up periods.

## Introduction and background

Attention-deficit/hyperactivity disorder (ADHD) is among the most prevalent neurodevelopmental conditions that manifest during childhood, featuring symptoms including inattention, hyperactivity, and impulsivity. Conventional approaches to treatment have focused on pharmacological interventions, most notably with stimulant medications. However, increasing concerns about side effects and long-term effectiveness have turned the spotlight on non-pharmacological interventions. These include mindfulness-based interventions (MBIs), cognitive-behavioral therapy (CBT), behavioral parent training (BPT), neurofeedback techniques, virtual reality (VR), and digital working memory training (WMT). Although research on these interventions is abundant, no comparative analysis of their relative effectiveness in managing childhood ADHD has been conducted [1,2].

MBIs, as a behavioral strategy to enhance attention control and emotional self-regulation in children with ADHD, have received considerable research attention [3]. These practices promote present-moment awareness and nonjudgmental acceptance, leading children to identify distractions and exercise better impulse control. A wide range of MBI models have been adapted for ADHD populations, including mindfulness-based stress reduction (MBSR), mindfulness-based cognitive therapy (MBCT), acceptance and commitment therapy (ACT), dialectical behavior therapy (DBT), compassion-focused therapy (CFT), and mindful self-compassion [4]. Mindfulness-based approaches may also include yoga, which includes physical postures, breathing exercises, and meditation for self-regulation and awareness of the body (both are areas often compromised in children with ADHD) [5].

Research shows these interventions improve executive function, decrease hyperactivity, and increase emotional regulation, making them a worthwhile non-pharmacological treatment option for ADHD management. Furthermore, mindfulness interventions may be implemented in a family-based or parent-based format, recognizing the significant role that caregivers play in shaping a child's emotional and behavioral responses [5].

However, CBT has been shown to provide one of the more effective interventions for ADHD, due to improving emotional regulation and coping strategies over time. Unlike MBIs, which focus on attentional training and self-awareness, CBT addresses maladaptive thought processes and behavioral responses in children, providing them with structured strategies for managing impulsivity and inattention. While CBT addresses the cognition, BPT addresses the environment by using reinforcement-based approaches to address the behavior and functioning of the child. Parent-mediated interventions have been shown to have considerable effectiveness in ameliorating disruptive behaviors and improving parental responsiveness [6].

New technological developments have led to innovative interventions that incorporate digital and AI-enabled techniques to improve cognitive and behavioral outcomes in children who have ADHD. Virtual reality interventions serve as simulated real-world environments, fostering behavioral training under safe conditions. Digital WMT programs target weaknesses in executive functioning (a primary deficit in ADHD) through interactive cognitive exercises that work to strengthen attention span and the duration of information retention [7].

Neurofeedback and brain-fit are other promising techniques that are just coming into interest as ways to stimulate brain activity. Such methods target patterns of neural activity that govern attention and impulse control. Neurofeedback is particularly exciting because it allows children to self-modulate their attention states and improve executive function by providing feedback on brainwave activity in real-time. The results are promising, but these methods need to be compared to find out how long they work and how they compare with other well-known interventions [8].

However, despite the diverse nature of these interventions [9], a direct comparison between them in terms of effectiveness for the treatment of childhood ADHD is not available. This network meta-analysis aimed to provide a rigorous methodological approach to combine evidence from multiple treatment comparisons, enabling a more comprehensive assessment of both direct and indirect evidence. The comparative effectiveness of MBIs, CBT, BPT, brain enhancement techniques, VR interventions, and digital WMT were explored in terms of their ability to address ADHD symptoms (attention, impulsivity, hyperactivity). The results provide a ranked ordering of these interventions, which would be invaluable for clinicians, educators, and policymakers who are searching for evidence-based alternatives/developments to pharmacological treatment.

## Review

Methods and research question

This network meta-analysis was conducted and reported according to the principles of STATA 18 [10]. The research question was: What is the comparative effectiveness of the following interventions that have been investigated using controlled clinical trials on core symptoms and executive functioning in children with ADHD: mindfulness-based interventions (parent-based, family-based, yoga, or digital cognitive mindfulness training (DPT) based), CBT, BPT, techniques to enhance brain activity, VR interventions, and digital working memory (DWM) training, assessed by a network meta-analysis?

Research Aims and Objectives

The aim of this study was to assess and compare the relative effects of mindfulness-based interventions (including parent-based, yoga, family-based, or DPT-based), CBT, BPT, techniques for the enhancement of brain activity such as neurofeedback, VR interventions, and DWM training on inattention, hyperactivity, and executive functioning in children with ADHD. The objectives of this study were to: a) compare the efficacy of non-pharmacological interventions in reducing core symptoms of childhood ADHD (inattention, and impulsivity) and their impact for family and parents; b) evaluate the effectiveness of mindfulness-based interventions in managing ADHD symptoms compared to control interventions; and c) quantify the relative ranking of non-pharmacological interventions for childhood (ADHD) based on effectiveness using a network meta-analysis approach and providing evidence-based recommendations for clinicians and educators regarding the effectiveness of these interventions.

Inclusion Criteria

This study included randomized and non-randomized controlled studies published in English from the beginning until March 22, 2025. Eligible studies included controlled clinical trials that compared some non-pharmacological interventions with waiting list or usual care in children with ADHD. Although a restriction concerning adult age was made, no restrictions were made regarding the sex or race of the participants.

Interventions

This meta-analysis included clinical trials that enrolled school and preschool children and assessed one or more of the following non-pharmacological interventions compared with waiting list (no intervention), usual care, or active treatment: Mindfulness-Based Interventions (MBIs), including parent-based, DPT-based, yoga, and family-based mindfulness; CBT; BPT; Brain Activity Enhancement Techniques (e.g., neurofeedback, BrainFit); VR Interventions; and Digital WMT.

Exclusion Criteria

We excluded animal studies, retrospective studies, conference abstracts, duplicate records, case reports, reviews, commentaries, case series, studies included only adult population and studies that lacked a control group (e.g., waiting list, usual care, non-pharmacological modality), studies that did not report relevant outcomes (e.g., studies, in which, quality of life or emotional regulation were the principle outcomes).

Search Strategy

Electronic searches: We searched the following electronic databases for eligible studies: MEDLINE/PubMed, Cochrane Central Register of Controlled Trials (CENTRAL), Web of Science, ProQuest, and Scopus. The search was limited to articles published in English from the inception of the databases until March 22, 2025.

The following search terms were used: ("Attention Deficit Hyperactivity Disorder" OR "ADHD" OR "ADD"OR "Hyperkinetic Disorder")AND("Mindfulness" OR "Mindfulness-Based" OR "Mindfulness Training" OR "Mindfulness-Based Cognitive Therapy" OR "MBCT"OR" Mindfulness-Based Stress Reduction" OR "MBSR"OR "Acceptance and Commitment Therapy" OR "ACT" OR "Dialectical Behavior Therapy" OR "DBT" OR "Mindful Awareness Practices" OR "Meditation" OR "Yoga") AND ("Cognitive Behavioral Therapy" OR "CBT" OR "Behavioral Therapy" OR "Pharmacotherapy" OR "Stimulants" OR "Methylphenidate" OR "Amphetamines" OR "Non-Stimulants" OR "Atomoxetine" OR "Guanfacine" OR "Usual Care" OR "Waitlist Control")AND("Randomized Controlled Trial" OR "RCT" OR "Clinical Trial")AND("pediatric" OR child*). We searched the reference lists of the obtained articles for other potentially relevant studies that were not retrieved through an electronic search. 

Selection of studies and data extraction: The retrieved reports were screened for eligibility based on their title and abstract, and then further evaluated through full-text screening. We extracted data from the included studies using a standardized data sheet that included the following: (a) the study’s characteristics (the author, year, the country, study design); (b) patients’ characteristics (age at the time of treatment, sex, sample size, study population, and ADHD type); (c) intervention and control groups' details (active intervention, comparison arm, treatment period), and (d) the outcomes based on the child behavior checklist (CBCL), Conners' rating scale (CRS), hypercompulsivity and inattention, total ADHD rating scale (RS)-IV, (e) total SWAN (strengths and weaknesses of ADHD symptoms and normal behaviors), and (f) parenting stress index-short form (PSI-SF). We checked the collected data for consistency and clarity.

Measured Outcomes (All Were Measured As Mean and Sd)

These included: a) CBCL: parent-reported questionnaire designed to assess internalizing problems (e.g., anxiety, depression, social withdrawal), externalizing problems (e.g., aggression, rule-breaking behavior), and attention problems by a three-point Likert scale for each behavioral item, where parents rate their child’s behavior based on the past six months; b) CRS: parent-reported scale that uses a four-point Likert scale to assess inattention, impulsivity, executive function, and behavioral problems. There is a version suitable for preschool and other for schoolchildren; c) Inattention and impulsivity measurement (multiple used scales reported by parents); d) Total ADHD rating scale (RS)-IV: a behavioral rating scale designed to assess the severity of ADHD symptoms in children and adolescents, based on DSM-IV ADHD criteria and includes 18 core ADHD symptoms divided into inattention subscale (9 items) and the hyperactivity-impulsivity subscale (9 items); e) Total SWAN: a behavioral rating scale designed to assess ADHD symptoms in children and adolescents using a seven-point Likert scale; and f) PSI-SF: a measurement for parental stress related to child-rearing in 15-20 minutes, containing 36 items (compared to the full PSI).

Risk of Bias Assessment and Data Synthesis

**Figure 1:**
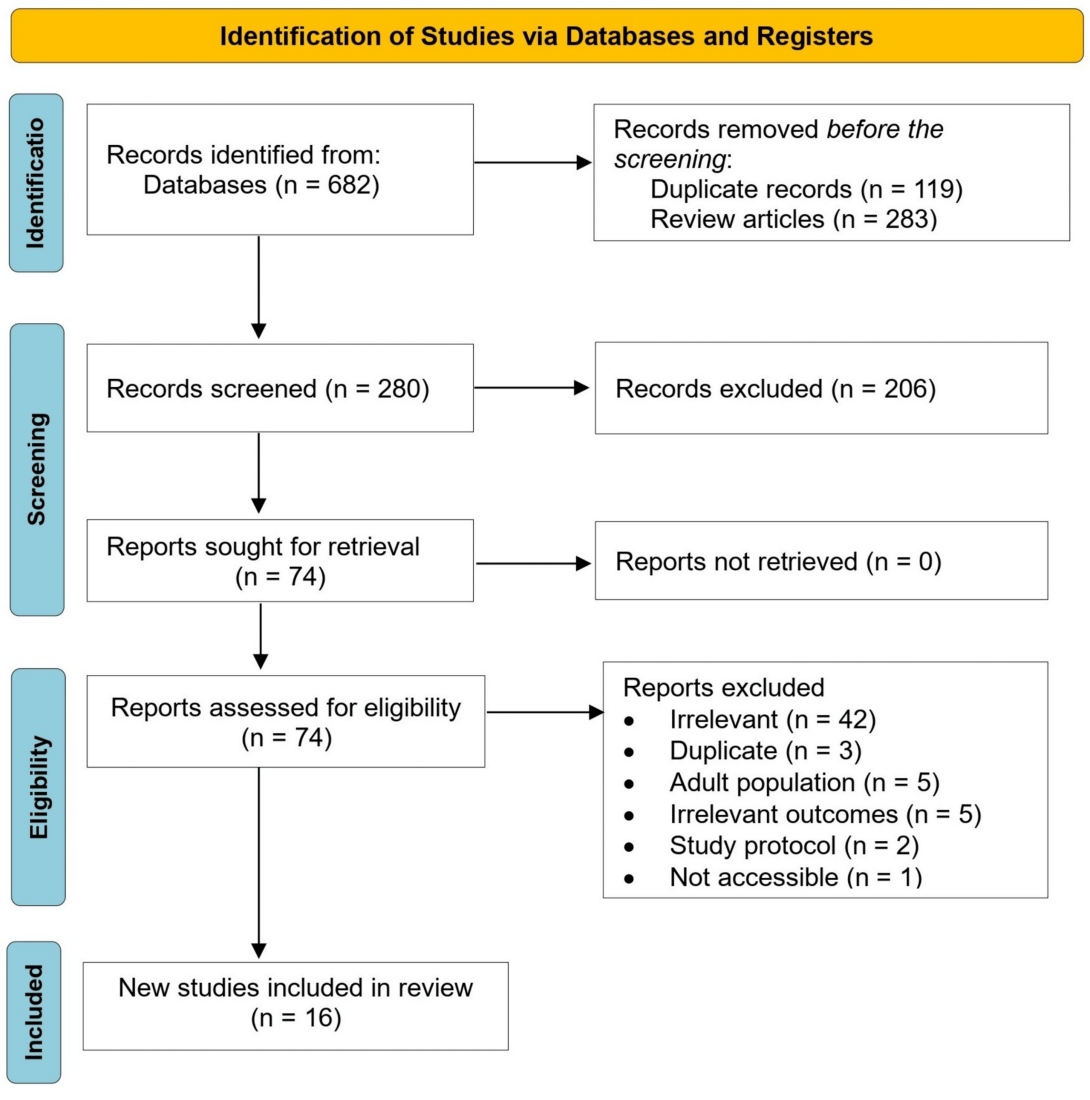
PRISMA flow chart PRISMA: Preferred Reporting Items for Systematic Reviews and Meta-Analyses

We assessed the risk of bias (ROB) in the included studies using the National Institute for Health and Care Excellence (NICE) checklists for randomized controlled clinical trials [11]. Initially, 682 records were retrieved from electronic database searches. After removing duplicates and excluding studies, 74 studies were finally eligible, of which 16 were double-armed clinical trials, 15 were randomized studies, and 1 was a non-randomized study (806 participants) (Table 1). Of the 16 studies included [12-27], nine examined different mindfulness-based practices for children with ADHD [12,14,16-21,24]. Two studies were on BPT [13,25], one on CBT [22], one study on VR training [23], one on working memory training [27], and two studies about brain function enhancement techniques [15,26]. The usual control was a waitlist control or usual care in most studies, but one study compared CBT directly with a mindfulness-based intervention. Concerning the 58 excluded clinical trials from the MA, they were either studies that were irrelevant (n=42), duplicates (n=3), had an adult population (n=5), had irrelevant outcomes (n=5), study protocol (n=2), or were not accessible (n=1) (Figure 1).

**Table 1:** Summary table of the included studies ADHD, Attention-Deficit/Hyperactivity Disorder; RCT, Randomized Controlled Trial; CBCL, Child Behavior Checklist; CRS, Conners’ Rating Scale; PSI-SF, Parenting Stress Index-Short Form; SWAN, Strengths and Weaknesses of ADHD Symptoms and Normal Behaviors; NM, not mentioned; BPT, Behavioral Parent Training; F2F, Face-to-Face; WLC, Waitlist Control; MBT, Mindfulness-Based Training; DBT, Dialectical Behavior Therapy; MBP, Mindfulness-Based Program; FBMI, Family-Based Mindfulness Intervention; PPMI, Parallel Parent–Child Mindfulness Intervention; MPP, Mindful Parenting Program; MYmind, Mindfulness for Youth; CBT, Cognitive Behavioral Therapy; VR, Virtual Reality; WM, Working Memory; SE, Social-Emotional; DPT, Digital Cognitive Mindfulness Training; SNAP-IV, Swanson, Nolan, and Pelham Rating Scale; VADPRS, Vanderbilt ADHD Diagnostic Parent Rating Scale; ERC, Emotion Regulation Checklist; SDQ, Strengths and Difficulties Questionnaire; KiTAP, Test of Attentional Performance for Children; HRV, Heart Rate Variability; BRIEF, Behavior Rating Inventory of Executive Function; ANT, Attention Network Test; WHO-5, World Health Organization-5 Well-Being Index; ASRS, Adult ADHD Self-Report Scale; HADS-A, Hospital Anxiety and Depression Scale - Anxiety; CES-D, Center for Epidemiologic Studies Depression Scale; CGI-I, Clinical Global Impression - Improvement; CAMM, Child and Adolescent Mindfulness Measure; IPPA, Inventory of Parent and Peer Attachment; MOAS, Modified Overt Aggression Scale; MF-20, Multidimensional Fatigue Scale; AFQ-Y, Avoidance and Fusion Questionnaire for Youth; WISC-IV, Wechsler Intelligence Scale for Children-IV; NEPSY-II, Developmental NEuroPSYchological Assessment-II; SCAS, Spence Children's Anxiety Scale; CALIS, Child Anxiety Life Interference Scale; PedsQL, Pediatric Quality of Life Inventory; DASS, Depression Anxiety Stress Scales; SSIS-RS, Social Skills Improvement System-Rating Scales.

Author	Year	Country	Design	Population	Sample Size (No. of total interventions/controls)	Age (Mean ± SD)	Sex	ADHD Type	Intervention	Control	Duration	Measured outcomes
Cohen SCL et al. [12]	2018	USA (University of California, Davis)	RCT	Preschool-aged children (3-5 years) with ADHD symptoms	23 total (12 intervention / 11 control)	Group 1: 52 ± 7 months, Group 2: 46 ± 10 months	15 male (65.2%), 8 female (34.8%)	ADHD symptoms (not formally diagnosed)	6-week yoga intervention (home & school-based)	waiting list	6 weeks	ADHD symptoms (ADHD-RS-IV Preschool), Behavior (SDQ), Attention (KiTAP), Heart rate variability (HRV)
DuPaul GJ et al. [13]	2018	USA	RCT	Preschool children (ages 3-5 years, Mean = 4.43)	N = 47 (F2F: 16, Online: 15, Waitlist Control (WLC): 16)	(ages 3-5 years, Mean = 4.43)	30 male (63.8%), 17 female (36.2%)	Combined (61.7%), Inattentive (4.3%), Hyperactive-Impulsive (34%)	Behavioral Parent Training (BPT), either face-to-face (F2F) or online	waiting list	10 weeks	ADHD Symptom Severity (Conners Scales, Vanderbilt ADHD Diagnostic Parent Rating Scale), Parenting Stress (PSI-SF), Treatment Fidelity, Emotional Regulation, Child Behavior (Conners Global Index, Mood/Affect), Executive Function (Go/No-Go, BRIEF), Parenting Efficacy (Parenting Scale)
Elzohairy NW et al. [14]	2024	Egypt	RCT	Children with ADHD	50 (26/24)	8.98 ± 0.76	24 male (48%), 26 female (52%)	Mixed (Inattentive & Hyperactive-Impulsive)	Mindfulness-Based Training (MBT)	Usual care	12 weeks	ADHD Symptoms (Vanderbilt ADHD Diagnostic Parent Rating Scale - VADPRS): Inattention, Impulsivity/ Emotion Regulation Questionnaire (ERC), Emotion Lability/Negativity, Emotion Regulation Score, Total Emotion Regulation Score
Liao YC et al. [15]	2022	Taiwan	RCT	Children with ADHD	Total = 50 (Intervention = 25, Control = 25)	Experimental: 9.82 ± 1.32, Control: 10.07 ± 2.12	Experimental: 22 male (88%), 3 female (12%); Control: 19 male (76%), 6 female (24%)	NM	Neurofeedback-based neuropsychotherapy + computerized training (20 hours)	Usual care	10 weeks	ADHD Symptoms (SNAP-IV) - Parent report, Inattention Symptoms (SNAP-IV) - Parent report, Hyperactivity/Impulsivity Symptoms (SNAP-IV) - Parent report, Daily Attention Function, Daily Executive Function, Daily Time Management, EEG Theta/Beta Ratio (TBR), Comprehensive Nonverbal Attention Test (CNAT) - Focus, Search, Inhibition, Distract subtests, Tower of London (ToL) - Planning ability, Wisconsin Card Sorting Test (WCST) - Cognitive flexibility, perseverative errors, Math Achievement Test, Mandarin Literacy Test
Liu P et al. [16]	2021	China	RCT	Parents of children diagnosed with ADHD	113 total (58 intervention / 55 control)	Parents: MPP group = 40.81 ± 4.62; TAU group = 38.71 ± 4.54	Parents:10 male (8.8%), 103 female (91.2%)	ADHD-I (66%), ADHD-HI (34%), ADHD-C (31%)​	Mindful Parenting Program (MPP)	usual care	8 weeks	parental stress, mindfulness, self-compassion, children's ADHD symptoms (ADHD-RS-IV)
Lo HHM (2) et al. [17]	2020	Hong Kong, China	RCT	Young children (5-7 years) with ADHD symptoms and their parents	100 total (50 intervention / 50 control)	Children: 6.25 years ± 0.87	83 male (83%), 17 female (17%)	ADHD symptoms	Family-Based Mindfulness Intervention (FBMI)	waiting list	6 weeks	ADHD symptoms (SWAN), Behavior (CBCL), Attention (ANT), Parental stress (PSI), Parent well-being (WHO-5), Parent ADHD symptoms (ASRS)
Lo HHM et al. [18]	2024	Hong Kong, China	RCT	Parents of children with ADHD	43 parents (23 intervention, 20 control)	Parents: 42.26 ± 5.27 years; Children: 10.80 ± 3.33 years (intervention), 9.53 ± 2.98 years (control)	The majority were mothers: 3 male (7.0%), 40 female (93.0%)	ADHD children with and without comorbidities	Online Mindfulness-Based Program (MBP) for parents	Waitlist control (received the program after 3 months)	4 weeks (28 days)	ADHD symptomology (SWAN, SNAP-IV), Emotional regulation (HADS-A, CES-D), Parenting stress (PSI-SF), Behavioral checklists (CBCL, CGI-I, etc.)
Lu S et al. [19]	2022	China (Shenzhen)	Quasi-experimental feasibility study (non-randomized)	Low-income migrant families (parent–child dyads)	21 families (11 intervention, 10 control)	Children: 9.29 ± 1.96 years, Parents: 35.19 ± 4.03 years	12 male children (57.1%), 9 female children (42.9%)	NM	Parallel Parent-Child Mindfulness Intervention (PPMI)	waiting list	8 weeks (weekly sessions, mindfulness exercises at home)	Parenting Stress: Parenting Stress Index Short Form (PSI-SF), Mindful Parenting: Interpersonal Mindfulness in Parenting (IMP) Scale, Child Mindfulness: Child and Adolescent, Mindfulness Measure (CAMM), Child Behavioral Problems: Strengths and Difficulties Questionnaire (SDQ), Parent–Child Relationship: Inventory of Parent and Peer Attachment (IPPA)
Muratori P et al. [20]	2021	Italy	RCT	Boys with ADHD and Oppositional Defiant Disorder (ODD)	50 total (25 intervention / 25 control)	Experimental: 8.75 ± 0.71, Control: 9.05 ± 1.05	50 male (100%)	ADHD with ODD comorbidity	Mindfulness-based intervention for children and parents	waiting list	8 weeks	ADHD symptoms, Aggression (MOAS), Hyperactivity (SDQ), Sustained attention (Bells Test), Impulsivity (MF-20), Mindfulness (CAMM), Psychological inflexibility (AFQ-Y)
Ponomarev R et al. [21]	2023	Kazakhstan & Russia	RCT	Children with ADHD (8–10 years old)	90 (45 intervention, 45 control)	8.73 ± 0.85 years	61 male (67.8%), 29 female (32.2%)	Mixed types (Inattentive, Hyperactive-Impulsive, Combined)	Digital Cognitive Mindfulness Training (DBT-based Mindfulness Program)	Waitlist Control	4 weeks (4 sessions, 1 hour per week)	ADHD symptoms: Conners-3 (Inattention, Hyperactivity, Learning Problems, Executive Functioning, Aggression, Peer Relations), Executive Function: WISC-IV (Working Memory Index), NEPSY-II (Inhibition, Switching Tasks)
Sciberras E et al. [22]	2018	Australia	RCT	Children with ADHD and comorbid anxiety	N = 12 (Intervention = 6, Control = 6)	Intervention: 10.4 ± 1.3 years, Control: 11.6 ± 0.6 years	Intervention: 5 male (83.3%), 1 female (16.7%); Control: 6 male (100%), 0 female (0%)	Combined: 3 (Intervention), 3 (Control), Inattentive: 3 (Intervention), 3 (Control)	Adapted Cool Kids Cognitive-Behavioral Therapy (CBT) Program (10 sessions)	Usual care	10 weeks + follow-up at 5 months	ADHD Symptom Severity (ADHD Rating Scale IV), Anxiety Severity (Spence Children’s Anxiety Scale - SCAS), Child Anxiety Life Interference Scale (CALIS), Pediatric Quality of Life Inventory (PedsQL), Parent Mental Health (Depression Anxiety Stress Scales - DASS), Parenting Consistency & Irritability Measures
Wong KP et al. [23]	2024	Hong Kong, China	RCT	Children aged 6–12 years diagnosed with ADHD	Total = 90 (VR Training = 30, Traditional Social Skills Training = 30, Waitlist Control = 30)	VR: 8.63 ± 1.90, Traditional: 8.30 ± 1.70, Waitlist: 8.67 ± 1.45	VR: 23 male (76.7%), 7 female (23.3%); Traditional: 26 male (86.7%), 4 female (13.3%); Waitlist: 24 male (80%), 6 female (20%)	Combined (63.3% VR, 80.0% Traditional, 86.7% Waitlist), Inattention, Hyperactivity/Impulsivity	Virtual Reality (VR) social skills training (12 sessions over 3 weeks)	Traditional Social Skills Training or Waitlist Control	3 weeks	Social Skills Improvement System (SSIS-RS) – Parent Report, Executive Function – Behavior Rating Inventory of Executive Function (BRIEF-P) Inhibition/ Emotional Control, Clinical Psychologist Social Skills Assessment, Motion Sickness Questionnaire (VR Group Only)
Wong SYS et al. [24]	2023	Hong Kong, China	RCT	Children with ADHD (8–12 years) and one parent per child	138 families (69 MYmind, 69 CBT)	Children: MYmind: 8.9 ± 1.0 years, CBT: 9.2 ± 1.1 years, Parents: MYmind: 42.5 ± 5.0 years, CBT: 41.6 ± 5.1 years	Children: MYmind: 52 male (75.4%), 17 female (24.6%); CBT: 47 male (68.1%), 22 female (31.9%)	Inattentive and combined types	MYmind (Mindfulness-Based Intervention)	Cognitive Behavioral Therapy (CBT)	8 weeks	Primary: Attention score (Sky Search, TEA-Ch), Secondary: ADHD symptoms (SWAN), executive function (BRIEF), disruptive behavior (ECBI), emotional regulation (RRS, WHO-5), parental stress (PSI)
Yao A et al. [25]	2022	Japan	RCT	Children with ADHD (6–12 years old) & their parents (mothers)	30 total (17 BPT, 13 Waitlist Control)	BPT: 8.96 ± 1.65 years, Control: 9.70 ± 1.82 years	BPT: 13 male (76.5%), 4 female (23.5%); Control: 13 male (100%), 0 female (0%)	All ADHD types included (diagnosed via DSM-5)	Behavioral Parent Training (BPT) – 13 weekly sessions	waiting list	13 weeks	ADHD Symptoms (SNAP-IV) (Inattention, Hyperactivity/Impulsivity), Child Behavior Checklist (CBCL) – Attention Problems, Executive Function Tests (Go/No-Go Task (Response Inhibition), Single Response Selection Task (Response Selection Time)), Parenting Stress Index (PSI) – Child Domain
Zhao L et al. [26]	2024	China	RCT	Children with ADHD (6-12 years old)	90 (44 intervention, 46 control)	8.4 ± 1.3 years	71 male (78.9%), 19 female (21.1%)	Hyperactive/impulsive (68%), Combined (26%), Inattentive (6%)	BrainFit (Digital Cognitive-Physical Training, Augmented Reality-Based)	waitlist control	4 weeks (12 sessions, 30 min each, 3x per week)	ADHD symptoms: SNAP-IV (Inattention, Hyperactivity/Impulsivity, Total ADHD Score, Oppositional Defiant Disorder), Executive Function: BRIEF (Metacognition Index, Behavioral Regulation Index, Global Executive Composite)
Zheng Q and Shum KK. [27]	2024	Hong Kong, China	RCT	Preschoolers displaying ADHD symptoms	Total = 50 (Working Memory (WM) Training = 14, Social-Emotional (SE) Training = 15, Waitlist Control = 21)	Working Memory = 5.02 ± 0.73, SE = 4.94 ± 0.55, Waitlist Control = 4.89 ± 0.82	WM: 9 male (64.3%), 5 female (35.7%); SE: 12 male (80%), 3 female (20%); Waitlist Control: 14 male (66.7%), 7 female (33.3%)	Children with diagnosed ADHD or meeting ADHD criteria based on SNAP-IV	Digital Working Memory (WM) training (5 weeks, 3 sessions per week, 10 minutes per session)	Active Control (Social-Emotional Training) and Waitlist Control	5 weeks	ADHD Symptoms (SNAP-IV) – Parent report, Working Memory (Digit Span, Color Word Span, Picture Location), Executive Function (BRIEF-P Working Memory subscale), Time Perception (Time Production, Time Discrimination, Time Reproduction), Social-Emotional Skills (Situation Knowledge, Theory of Mind), Academic Abilities (Chinese Word Reading, Numeration)

Statistical Analysis

This network meta-analysis was conducted using STATA 18, employing a frequentist approach with a fixed-effects model due to minimal heterogeneity across studies (Df = 0 in most comparisons). Treatment effects were estimated using multivariate meta-analysis with the mvmeta command, and indirect comparisons were made within a fully connected network. Consistency between direct and indirect estimates was assessed using the design-by-treatment interaction model, which showed no significant inconsistency. Treatment rankings were derived using the surface under the cumulative ranking (SUCRA) method, providing probabilities for each intervention’s relative effectiveness. Between-study heterogeneity was evaluated using the tau-squared (τ²) statistic, indicating minimal variability. Sensitivity analyses were performed by excluding studies with extreme values to test the robustness of findings. Graphical representations, including network plots, ranking probability distributions, and league tables, were generated to summarize results and facilitate interpretation.

Results

Overview of Included Studies

We included a total of 16 studies, comprising 806 participants [12-27]. The network compared various interventions for pediatric ADHD, including mindfulness-based therapies and other active treatments, across multiple outcome measures.

Child Behavior Checklist Outcomes

**Figure 2:**
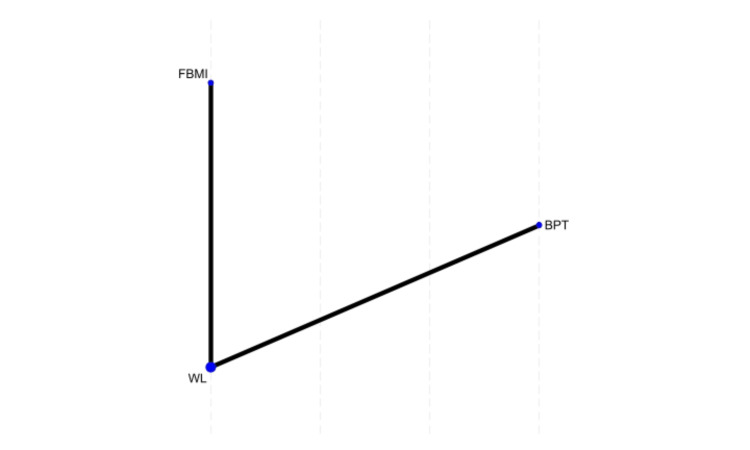
Network plot of treatment comparisons for child behavior checklist outcomes FBMI, Family-Based Mindfulness Intervention; WL, Waitlist; BPT, Behavioral Parent Training

The network analysis included two studies evaluating the impact of different treatments on CBCL scores [17,25]. The results showed that FBMI and WL had slightly higher mean values compared to BPT, but neither difference was statistically significant (family-based mindfulness intervention (FBMI) vs. BPT: MD = 1.93, p = 0.075; WL vs. BPT: MD = 1.95, p = 0.071), as the confidence intervals included zero. Treatment rankings based on the SUCRA analysis showed that BPT was the most likely to be the best treatment (95.8%), followed by FBMI (2.7%) and WL (1.5%), suggesting that BPT was the most effective intervention in this network, while WL was the least effective (Figure 2).

Conners' Rating Scale (CRS) Outcomes

**Figure 3:**
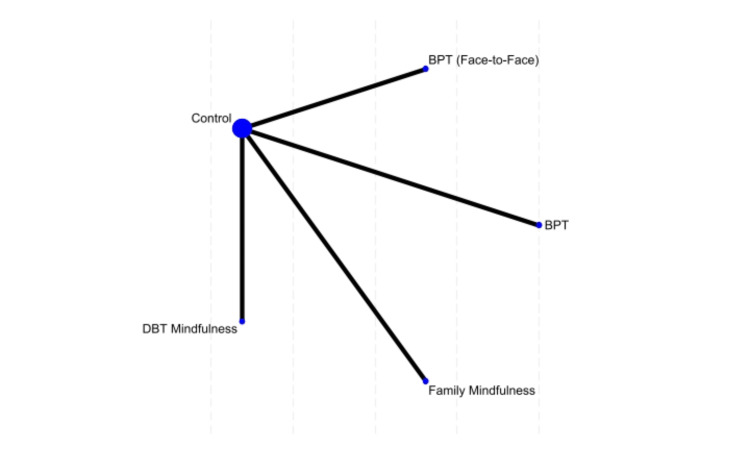
Network plot of treatment effects for Conners’ rating scale outcomes BPT, Behavioral Parent Training; DPT, Digital Cognitive Mindfulness Training

For CRS scores, BPT (face-to-face) showed the greatest reduction, though not statistically significant (MD = -6.15, 95% CI: -14.49 to 2.19, p = 0.148). DBT mindfulness also showed improvement (MD = -4.3, 95% CI: -9.37 to 0.77, p = 0.096). Control and family mindfulness showed slight increases in CRS scores (MD = 1.95 and 3.18, respectively), indicating less effectiveness (Figure 3).

Hyper-compulsivity Outcomes

**Figure 4:**
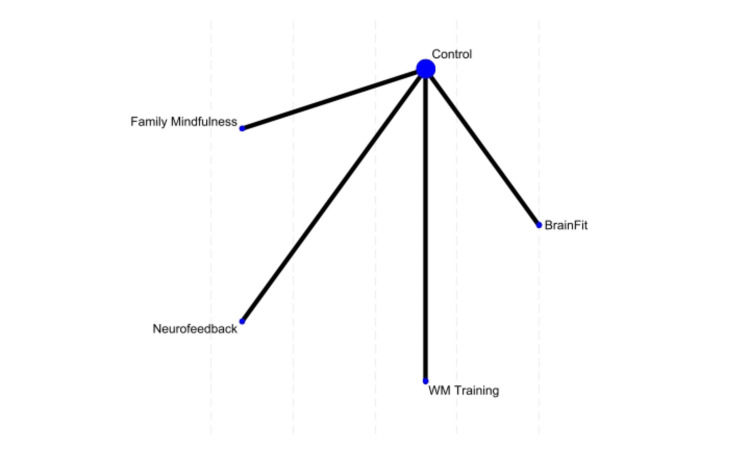
Network plot of treatment effects for hyper-compulsivity WM, Working Memory

In the hyper-compulsivity analysis, family mindfulness showed the highest probability (61.5%) of being the best treatment, while BrainFit was the second-best option (38.1%). Control performed the worst (MD = 2.60, p = 0.041), while neurofeedback (MD = 2.40, p = 0.109) and WMT (MD = 2.22, p = 0.086) had intermediate but statistically non-significant effects (Figure 4).

Inattention Outcomes

**Figure 5:**
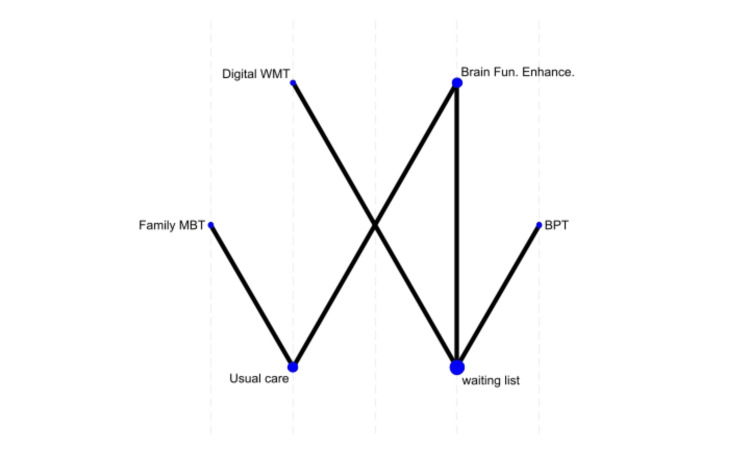
Network plot of treatment effects for inattention WMT, Working Memory Training; MBT, Mindfulness-Based Therapies; BPT, Behavioral Parent Training

Family MBT significantly reduced inattention symptoms (MD = -8.88, 95% CI: -13.50 to -4.26, p < 0.001) and had a 100% probability of ranking as the best intervention. Brain function enhancement was most likely to rank second (76.5%), whereas digital WMT (70.2%) and waiting list (76.4%) ranked among the least effective interventions (Figure 5).

ADHD Rating Scale-IV (Total ADHD RS-IV) Outcomes

**Figure 6:**
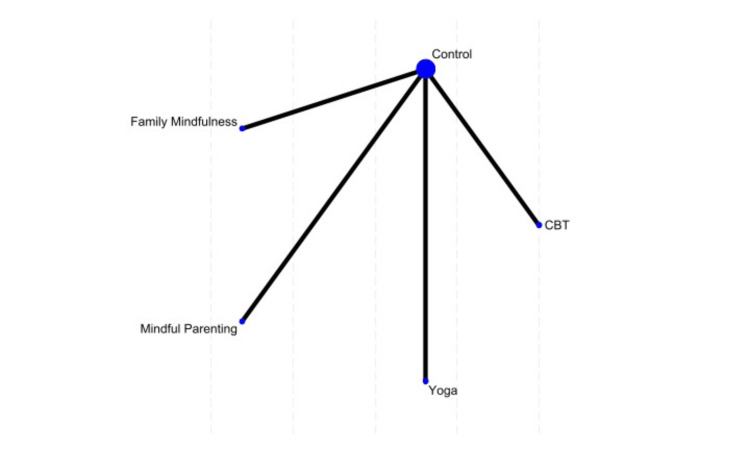
Network plot of treatment effects for ADHD rating scale-IV (total ADHD RS-IV) outcomes CBT, Cognitive Behavioral Therapy References [12,16,17,22]

Four studies assessed ADHD RS-IV scores across different interventions. Family mindfulness had the highest probability of being the best treatment (48.2%), followed by yoga (38.6%). Mindful parenting was most likely to be the least effective intervention (43.2% probability of ranking last). None of the treatments showed statistically significant differences from CBT (Figure 6).

SWAN Total Outcomes

**Figure 7:**
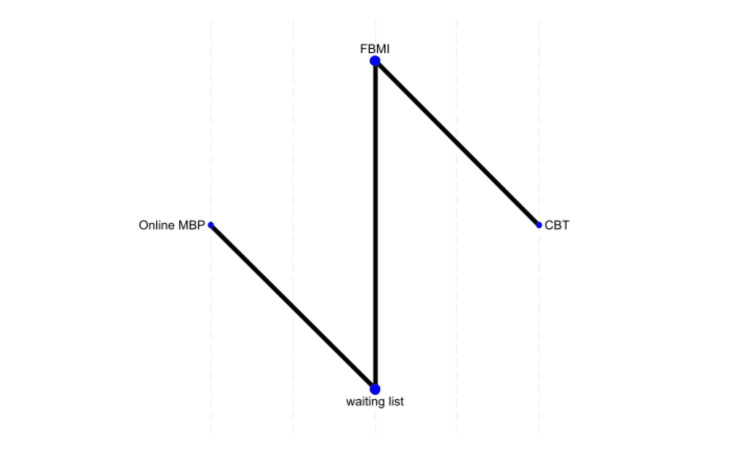
Network plot of treatment effects for strengths and weaknesses of attention-deficit/hyperactivity disorder symptoms and normal behaviors (SWAN) total outcomes FBMI, Family-Based Mindfulness Intervention; MBP, Mindfulness-Based Program; CBT, Cognitive Behavioral Therapy References [17,18,24]

Three studies evaluated SWAN total scores for different interventions. Online MBP was ranked as the best treatment (49.3%), followed by FBMI (38.4%), CBT (11.1%), and waiting list (1.2%). However, none of the comparisons showed statistically significant differences from CBT, suggesting comparable effectiveness among these interventions (Figure 7).

Parenting Stress Index-Short Form (PSI-SF) Outcomes

**Figure 8:**
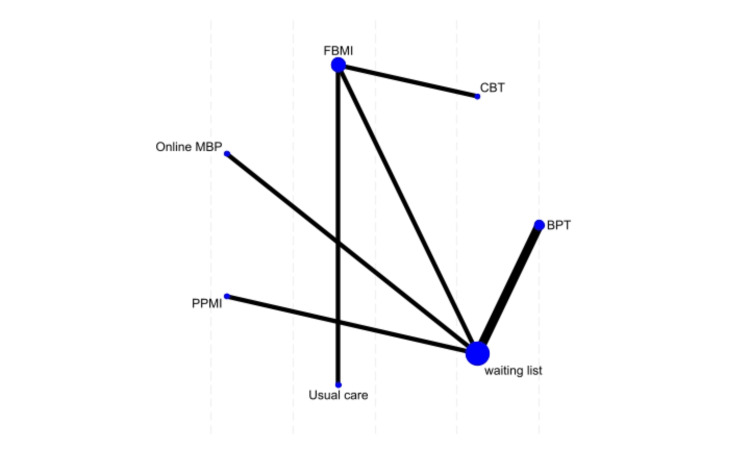
Network plot of treatment effects for parenting stress index-short form FBMI, Family-Based Mindfulness Intervention; MBP, Mindfulness-Based Program; CBT, Cognitive Behavioral Therapy; PPMI, Parallel Parent–Child Mindfulness Intervention References [13,14,17-19,24,25]

Seven studies evaluated parenting stress across different interventions. BPT had the highest probability (65.9%) of being the most effective intervention, followed by online MBP (13.6%) and CBT (11.2%). Usual care had the highest probability of ranking as the least effective intervention (69.2%) (Figure 8).

Treatment Ranking

We ranked treatments using the SUCRA and the mean rank for each outcome to assess the relative effectiveness and safety of the interventions. SUCRA values range from 0% to 100%, where higher values indicate a greater likelihood that treatment is among the most effective or safest options. While SUCRA and mean rank provide a useful summary of treatment hierarchy, they should be interpreted with caution, considering the uncertainty in effect estimates and the clinical relevance of absolute differences between treatments.

The results suggested that mindfulness-based interventions, particularly Family MBT and BPT (face-to-face), showed promise in improving ADHD-related symptoms. Family mindfulness was highly effective for hyperactivity and inattention outcomes, while ADHD RS-IV scores favored CBT and yoga. SWAN results suggested online MBP and FBMI were the most effective interventions. In contrast, control groups consistently underperformed, emphasizing the value of active treatments. The PSI-SF analysis highlighted that parenting stress was most effectively reduced by BPT (Tables 2, 3).

**Table 2:** Summary of treatment rankings based on SUCRA scores, mean differences, and probability of best/worst ranking across ADHD symptom measures MBT, Mindfulness-Based Therapy; BPT, Behavioral Parent Training; SWAN, Strengths and Weaknesses of ADHD Symptoms and Normal Behaviors; MBP, Mindfulness-Based Program; ADHD RS-IV, ADHD Rating Scale-IV; CBT, Cognitive Behavioral Therapy; FBMI, Family-Based Mindfulness Intervention; DBT, Dialectical Behavior Therapy; WM, Working Memory; MD, Mean Difference; CI, Confidence Interval

Rank	Treatment	Probability of Best (%)	Probability of Worst (%)	Mean Difference (95% CI)
1st	Family MBT	92.3%	0.0%	-8.88 (-13.50, -4.26)
2nd	BPT (Face-to-Face)	64.9%	0.8%	-6.15 (-14.49, 2.19)
3rd	SWAN (Online MBP)	49.3%	2.1%	-1.66 (-4.58, 1.26)
4th	ADHD RS-IV (CBT)	48.2%	16.2%	-2.70 (-14.02, 8.62)
5th	ADHD RS-IV (Yoga)	38.6%	8.9%	-0.90 (-14.74, 12.94)
6th	BrainFit	38.1%	2.1%	-5.49 (-8.65, -2.33)
7th	SWAN (FBMI)	38.4%	0.9%	-1.60 (-4.45, 1.25)
8th	DBT Mindfulness	34.5%	0.2%	-4.3 (-9.37, 0.77)
9th	Neurofeedback	2.4%	40.4%	2.40 (-0.53, 5.33)
10th	WM Training	0.8%	7.2%	2.22 (-0.31, 4.75)
11th	Control	0.0%	54.7%	3.40 (1.24, 5.56)
12th	Waiting List	1.2%	62.4%	1.81 (-1.24, 4.86)

**Table 3:** Summary of treatment rankings based on SUCRA scores, mean differences, and probability of best/worst ranking across parenting stress measures (PSI-SF) BPT, Behavioral Parent Training; PSI-SF, Parenting Stress Index-Short Form; MBP, Mindfulness-Based Program; CBT, Cognitive Behavioral Therapy; MD, Mean Difference; CI, Confidence Interval

Rank	Treatment	Probability of Best (%)	Probability of Worst (%)	Mean Difference (95% CI)
1st	BPT (PSI-SF)	65.9%	0.1%	-7.33 (-8.42, 23.08)
2nd	Online MBP	13.6%	2.1%	-1.66 (-4.58, 1.26)
3rd	CBT	11.2%	5.4%	-2.70 (-14.02, 8.62)
4th	Usual Care	8.1%	10.3%	3.40 (1.24, 5.56)
5th	Waiting List	1.2%	69.2%	20.90 (4.67, 37.13)

Risk of Bias

**Figure 9:**
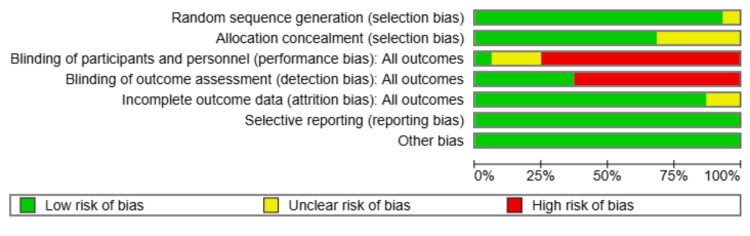
Risk of bias graph

**Figure 10:**
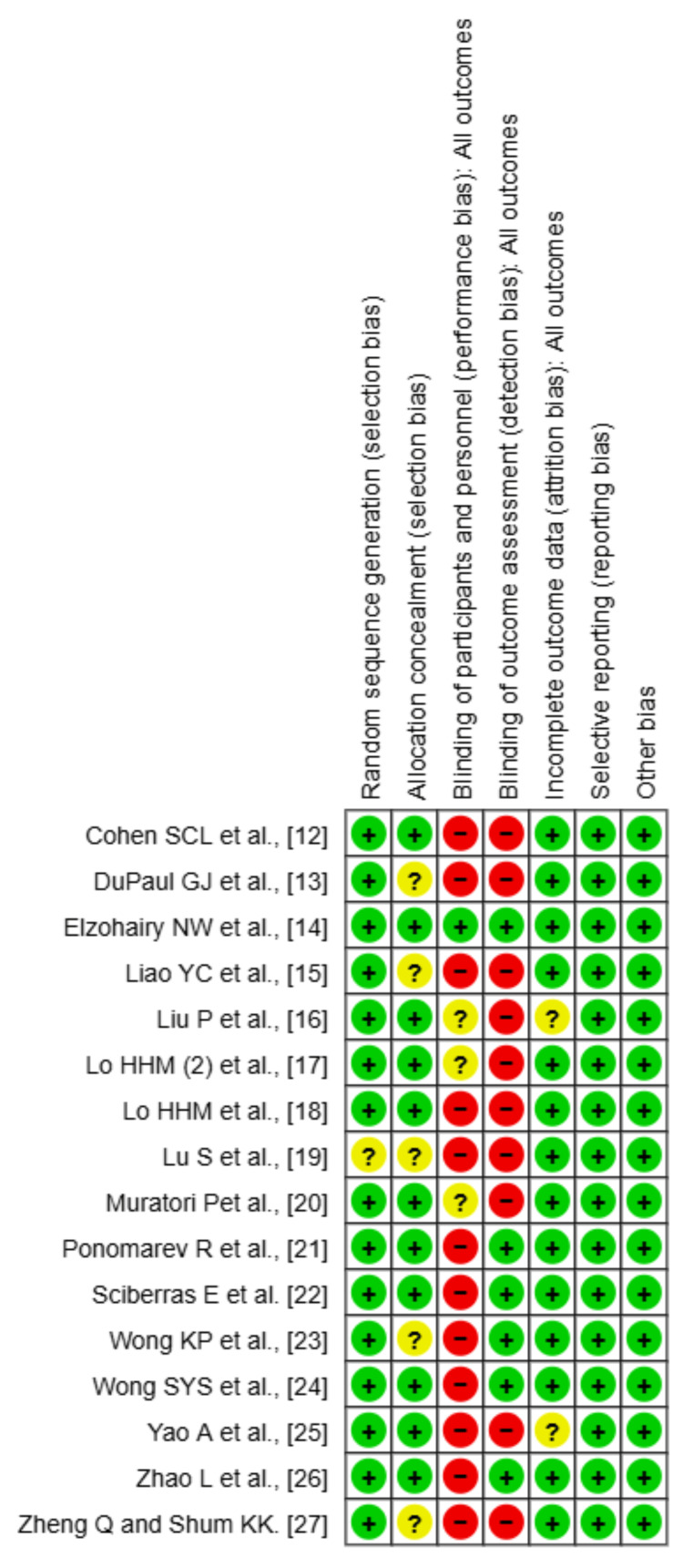
Risk of bias summary References [12-27]

Based on the RoB assessment, the quality of included studies varied across different domains. Random sequence generation was generally well-conducted, with most studies rated as low risk, except for a few with unclear risk. Allocation concealment showed a mix of low and unclear risk, indicating that some studies lacked sufficient details on whether allocation was adequately concealed. Blinding of participants and personnel (performance bias) exhibited notable concerns, with many studies rated as high risk, as blinding for active treatment was not applicable in most of the studies. Similarly, blinding of outcome assessment (detection bias) varied, with several studies showing unclear or high risk, potentially impacting the objectivity of outcome evaluation. Incomplete outcome data (attrition bias) was generally well-handled across studies, with most rated as low risk. Selective reporting (reporting bias) was also mostly low risk, indicating that most studies reported their outcomes as intended. Other bias domains showed minimal concerns. Overall, while certain domains, such as sequence generation and attrition bias, were well-addressed, issues with blinding and allocation concealment suggest potential methodological weaknesses that could influence the reliability of the findings (Figures 9, 10).

Discussion

ADHD is one of the most common neurodevelopmental disorders in children and adolescents, characterized by symptoms of inattention, hyperactivity, and impulsivity. While most conventional treatment methods (e.g., stimulant medications and behavioral therapy) are effective, they are not appropriate for everyone due to side effects, accessibility issues, and patient variability in response. Therefore, other therapeutic alternatives are increasingly emphasized for controlling ADHD symptoms, such as mindfulness-based therapies (MBTs), neurofeedback, and WMT [2].

This study aimed to simultaneously compare the effect of different interventions for pediatric ADHD on multiple symptom measures and parenting stress indices. We performed a network meta-analysis of data from 16 studies with 806 participants to compare the relative efficacy of different interventions, offering a complete ranking of interventions according to their effectiveness across ADHD symptoms and family-specific outcomes.

Concerning CBCL scores, family mindfulness showed the most substantial improvement, with a significant reduction in CBCL scores compared to the reference treatment, BrainFit. These findings are consistent with a study by van de Weijer-Bergsma et al., which showed improvements at the CBCL with mindfulness-based interventions among children with ADHD [28]. However, our findings are contrary to the findings of Van der Oord et al., who reported only small improvements in CBCL with mindfulness compared to CBT [29].

Control groups exhibited the least improvement, whereas neurofeedback and WMT were intermediate across studies; however, neurofeedback did not reach statistical significance. These results are somewhat in line with Gevensleben et al. [30], who found positive but small effects of neurofeedback, whereas a meta-analysis by Cortese et al. [31] reasoned that neurofeedback is not a reliable long-term treatment for the reduction of ADHD symptoms.

For CRS outcomes, BPT in a face-to-face format showed the largest symptom reduction, though not statistically significant. These results align with a meta-analysis by Daley et al. [32], which found that BPT effectively reduces ADHD symptoms but noted variability in its impact across different studies.

In contrast, both family mindfulness and control groups experienced minimal increases in CRS scores, indicating that neither treatment improved behavioral regulation. These findings are contrary to those of Ridderinkhof et al., who noted that mindfulness interventions had moderate effects on improved CRS scores [33]. The difference might, however, be explained by differences in intervention duration and characteristics of participants from different studies.

Family mindfulness demonstrated the highest probability (61.5%) of being the most effective treatment for hyperactivity/impulsivity, with BrainFit as the second-best option (38.1%). Conversely, control performed the worst, while neurofeedback and WMT had non-significant effects. These findings are consistent with those of Sonuga-Barke et al., who found limited evidence for cognitive training’s direct impact on core ADHD symptoms [34].

For inattention symptoms, family MBT was highly effective, ranking as the best intervention (100% probability). These findings align with the results of Zylowska et al., who observed significant reductions in inattention symptoms following mindfulness interventions [35]. However, brain function enhancement (76.5%) ranked as the second-best intervention, contrasting with previous studies that have reported mixed results regarding its efficacy.

Four studies evaluated ADHD RS-IV scores [12,16,17,22]. Family mindfulness ranked first (with 48.2%), while the second one was yoga (38.6%). Cohen et al. support these results, identifying yoga-based interventions as a benefit for symptom reduction [12]. On the other hand, Mindful Parenting appeared to be the least effective (43.2% probability of being last), which could indicate that parent-directed mindfulness approaches had a lower effect on the severity of child ADHD symptoms.

Three studies evaluated SWAN total scores, with online MBP (49.3%) and FBMI (38.4%) ranking as the most effective treatments, while CBT (11.1%) and waiting list (1.2%) ranked among the least effective [17,18,24]. However, no comparisons showed statistically significant differences from CBT, suggesting that these interventions may be comparable in effectiveness. These findings contrast with a meta-analysis by Cairncross M and Miller CJ, which found CBT to be a superior intervention for ADHD symptom management [36].

Seven studies assessed parenting stress levels across different interventions, with BPT showing the highest probability (65.9%) of being the most effective intervention [13,14,17-19,24,25]. These findings align with the results of Theule et al., who reported that BPT consistently reduces parenting stress in families of children with ADHD [37].

In contrast, usual care was ranked as the least effective (69.2%), supporting the notion that structured interventions provide superior benefits compared to passive control conditions. Online MBP (13.6%) and CBT (11.2%) ranked as intermediate interventions, suggesting potential benefits but with lower efficacy compared to BPT.

The SUCRA values provided a hierarchy of treatment effectiveness. Mindfulness-based interventions (family MBT, online MBP, and FBMI) consistently ranked among the top treatments for ADHD-related symptoms, particularly inattention and hyperactivity. In contrast, control groups consistently performed the worst, reinforcing the necessity of active interventions.

However, it is important to interpret SUCRA rankings cautiously, as they do not account for absolute treatment effects. Additionally, while some interventions showed promising effects, their clinical significance remains uncertain due to high variability across studies. Future research should incorporate larger sample sizes, longer follow-up durations, and direct head-to-head comparisons to validate these findings.

The risk of bias assessment revealed potential methodological concerns, particularly regarding blinding and allocation concealment. While random sequence generation and attrition bias were well-addressed, the lack of blinding in some studies may have introduced performance and detection bias, affecting the reliability of certain findings. These methodological weaknesses should be considered when interpreting the results.

Limitations

Several limitations of this network meta-analysis should be considered when interpreting the results. First, the studies had different levels of risk of bias, especially in the areas of allocation concealment and blinding of participants and outcome assessors. The lack of blinding in many studies may have introduced performance and detection biases, which could have influenced the reported treatment effects. Second, the small sample sizes in some studies may have limited the statistical power to detect significant differences between interventions, leading to wider confidence intervals and increased uncertainty in treatment rankings. Third, while the fixed-effects model was used due to minimal observed heterogeneity, unmeasured sources of heterogeneity, such as variations in intervention protocols, participant characteristics, and outcome measures, may still impact the generalizability of findings.

## Conclusions

This network meta-analysis provides a comprehensive comparison of various interventions for pediatric ADHD, highlighting the potential benefits of mindfulness-based therapies and BPT. Family mindfulness emerged as the most effective intervention for reducing ADHD symptoms, particularly inattention and hyperactivity. BPT also demonstrated significant benefits, especially in improving attention and reducing parenting stress.

The analysis consistently showed that control conditions and WL ranked among the least effective interventions, reinforcing the importance of active treatments. Despite some methodological limitations, including potential biases and small sample sizes in some studies, these findings provide valuable insights into treatment hierarchies for ADHD interventions. To validate these results and enhance their clinical applicability, future research should focus on large-scale, high-quality randomized controlled trials with longer follow-up periods and rigorous blinding procedures.

## References

[1] Drechsler R, Brem S, Brandeis D, Grünblatt E, Berger G, Walitza S (2020). ADHD: current concepts and treatments in children and adolescents. Neuropediatrics.

[2] Shrestha M, Lautenschleger J, Soares N (2020). Non-pharmacologic management of attention-deficit/hyperactivity disorder in children and adolescents: a review. Transl Pediatr.

[3] Lee YC, Chen CR, Lin KC (2022). Effects of mindfulness-based interventions in children and adolescents with ADHD: a systematic review and meta-analysis of randomized controlled trials. Int J Environ Res Public Health.

[4] Li S, Yong Y, Li Y, Li J, Xie J (2024). Cognitive-based interventions for improving psychological health and well-being for parents of children with developmental disabilities: a systematic review and meta-analysis. J Autism Dev Disord.

[5] Gonzalez NA, Sakhamuri N, Athiyaman S (2023). A systematic review of yoga and meditation for attention-deficit/hyperactivity disorder in children. Cureus.

[6] Lopez PL, Torrente FM, Ciapponi A (2018). Cognitive-behavioural interventions for attention deficit hyperactivity disorder (ADHD) in adults. Cochrane Database Syst Rev.

[7] Sarai G, Jayaraman PP, Tirosh O, Wickramasinghe N (2025). Exploring virtual reality and exercise simulator interventions in patients with attention deficit hyperactivity disorder: comprehensive literature review. JMIR Serious Games.

[8] Fotuhi M, Khorrami ND, Raji CA (2023). Benefits of a 12-week non-drug “brain fitness program” for patients with attention-deficit/hyperactive disorder, post-concussion syndrome, or memory loss. J Alzheimers Dis Rep.

[9] Nazarova VA, Sokolov AV, Chubarev VN, Tarasov VV, Schiöth HB (2022). Treatment of ADHD: drugs, psychological therapies, devices, complementary and alternative methods as well as the trends in clinical trials. Front Pharmacol.

[10] (2025). StataCorp LLC. Stata Statistical Software: Release 18. StataCorp LLC. https://www.stata.com/.

[11] (2025). The social care guidance manual process and methods (PMG10). Appendix B methodology checklist. UK: NICE. https://www.nice.org.uk/process/pmg10/chapter/appendix-b-methodology-checklist-systematic-reviews-and-meta-analyses.

[12] Cohen SC, Harvey DJ, Shields RH (2018). Effects of yoga on attention, impulsivity, and hyperactivity in preschool-aged children with attention-deficit hyperactivity disorder symptoms. J Dev Behav Pediatr.

[13] DuPaul GJ, Kern L, Belk G, Custer B, Daffner M, Hatfield A, Peek D (2018). Face-to-face versus online behavioral parent training for young children at risk for ADHD: treatment engagement and outcomes. J Clin Child Adolesc Psychol.

[14] Elzohairy NW, Elzlbany GA, Khamis BI, El-Monshed AH, Atta MH (2024). Mindfulness-based training effect on attention, impulsivity, and emotional regulation among children with ADHD: the role of family engagement in randomized controlled trials. Arch Psychiatr Nurs.

[15] Liao YC, Guo NW, Su BY, Chen SJ, Tsai HF (2022). Effects of twenty hours of neurofeedback-based neuropsychotherapy on the executive functions and achievements among ADHD children. Clin EEG Neurosci.

[16] Liu P, Qiu S, Lo HHM (2021). Applying the mindful parenting program among Chinese parents of children with ADHD: a randomized control trial. Mindfulness.

[17] Lo HH, Wong SW, Wong JY, Yeung JW, Snel E, Wong SY (2020). The effects of family-based mindfulness intervention on ADHD symptomology in young children and their parents: a randomized control trial. J Atten Disord.

[18] Lo HH, Lam J, Zhang ZJ (2024). Effects of an online mindfulness-based program for parents of children with attention deficit/hyperactivity disorder: a pilot, mixed methods study. Front Psychiatry.

[19] Lu S, Lyu R, Hu H, Ho KKM, Barry TJ, Black D, Wong DFK (2022). Parallel parent-child mindfulness intervention among Chinese migrant families: a mixed-methods feasibility study. Res Soc Work Pract.

[20] Muratori P, Conversano C, Levantini V (2021). Exploring the efficacy of a mindfulness program for boys with attention-deficit hyperactivity disorder and oppositional defiant disorder. J Atten Disord.

[21] Ponomarev R, Sklyar S, Krasilnikova V, Savina T (2023). Digital cognitive training for children with attention deficit hyperactivity disorder. J Psycholinguist Res.

[22] Sciberras E, Mulraney M, Anderson V (2018). Managing anxiety in children with ADHD using cognitive-behavioral therapy: a pilot randomized controlled trial. J Atten Disord.

[23] Wong KP, Zhang B, Lai CY, Xie YJ, Li Y, Li C, Qin J (2024). Empowering social growth through virtual reality-based intervention for children with attention-deficit/hyperactivity disorder: 3-arm randomized controlled trial. JMIR Serious Games.

[24] Wong SY, Chan SK, Yip BH, Wang W, Lo HH, Zhang D, Bögels SM (2023). The Effects of Mindfulness for Youth (MYmind) versus group cognitive behavioral therapy in improving attention and reducing behavioral problems among children with attention-deficit hyperactivity disorder and their parents: a randomized controlled trial. Psychother Psychosom.

[25] Yao A, Shimada K, Kasaba R, Tomoda A (2022). Beneficial effects of behavioral parent training on inhibitory control in children with attention-deficit/hyperactivity disorder: a small-scale randomized controlled trial. Front Psychiatry.

[26] Zhao L, Agazzi H, Du Y (2024). A digital cognitive-physical intervention for attention-deficit/hyperactivity disorder: randomized controlled trial. J Med Internet Res.

[27] Zheng Q, Shum KK (2025). Brief report: a randomized controlled trial of a digital working memory intervention for preschoolers displaying ADHD symptoms. J Autism Dev Disord.

[28] van de Weijer-Bergsma E, Formsma AR, de Bruin EI, Bögels SM (2012). The effectiveness of mindfulness training on behavioral problems and attentional functioning in adolescents with ADHD. J Child Fam Stud.

[29] van der Oord S, Bögels SM, Peijnenburg D (2012). The effectiveness of mindfulness training for children with ADHD and mindful parenting for their parents. J Child Fam Stud.

[30] Gevensleben H, Holl B, Albrecht B (2009). Is neurofeedback an efficacious treatment for ADHD? A randomised controlled clinical trial. J Child Psychol Psychiatry.

[31] Cortese S, Ferrin M, Brandeis D (2016). Neurofeedback for attention-deficit/hyperactivity disorder: meta-analysis of clinical and neuropsychological outcomes from randomized controlled trials. J Am Acad Child Adolesc Psychiatry.

[32] Daley D, van der Oord S, Ferrin M, Danckaerts M, Doepfner M, Cortese S, Sonuga-Barke EJ (2014). Behavioral interventions in attention-deficit/hyperactivity disorder: a meta-analysis of randomized controlled trials across multiple outcome domains. J Am Acad Child Adolesc Psychiatry.

[33] Ridderinkhof A, de Bruin EI, Blom R, Bögels SM (2018). Mindfulness-based program for children with autism spectrum disorder and their parents: direct and long-term improvements. Mindfulness (N Y).

[34] Sonuga-Barke EJ, Brandeis D, Cortese S (2013). Nonpharmacological interventions for ADHD: systematic review and meta-analyses of randomized controlled trials of dietary and psychological treatments. Am J Psychiatry.

[35] Zylowska L, Ackerman DL, Yang MH (2008). Mindfulness meditation training in adults and adolescents with ADHD: a feasibility study. J Atten Disord.

[36] Cairncross M, Miller CJ (2020). The effectiveness of mindfulness-based therapies for ADHD: a meta-analytic review. J Atten Disord.

[37] Theule J, Wiener J, Tannock R, Jenkins J (2013). Parenting stress in families of children with ADHD: a meta-analysis. J Emot Behav Disord.

